# Pre-operative planning for mandibular reconstruction - A full digital planning workflow resulting in a patient specific reconstruction

**DOI:** 10.1186/1758-3284-3-45

**Published:** 2011-10-03

**Authors:** Harald Essig, Majeed Rana, Horst Kokemueller, Constantin von See, Martin Ruecker, Frank Tavassol, Nils-Claudius Gellrich

**Affiliations:** 1 Department of Oral and Maxillofacial Surgery, Hannover Medical School, Hannover, Germany

**Keywords:** Mandibular reconstruction, backward planning, patient specific implant, computer-assisted surgery

## Abstract

**Objectives:**

Reconstruction of large mandiblular defects following ablative oncologic surgery could be done by using vascularized bone transfer or, more often, primarily with simultaneous or delayed bone grafting, using load bearing reconstruction plates. Bending of these reconstruction plates is typically directed along the outer contour of the original mandible. Simultaneously or in a second operation vascularized or non-vascularized bone is fixed to the reconstruction plate. However, the prosthodontic-driven backward planning to ease bony reconstruction of the mandible in terms of dental rehabilitation using implant-retained overdentures might be an eligible solution. The purpose of this work was to develop, establish and clinically evaluate a novel 3D planning procedure for mandibular reconstruction.

**Materials and methods:**

Three patients with tumors involving the mandible, which included squamous cell carcinoma in the floor of the mouth and keratocystic odontogenic tumor, were treated surgically by hemimandibulectomy.

**Results:**

In primary alloplastic mandible reconstruction, shape and size of the reconstruction plate could be predefined and prebent prior to surgery.

**Clinical relevance:**

This study provides modern treatment strategies for mandibular reconstruction.

## Introduction

Indications for partial and total mandibulectomy include malignancies especially squamous cell carcinomas (SCC), benign tumors such as ameloblastomas and sequelae of radiotherapy such as osteoradionecrosis. Defect size in ablative surgery mainly depends on dignity and dimensions of the pathology. Following mandibulectomy, reconstruction of the mandible is mainly done with vascularized bone grafts or alloplastic materials (reconstruction plates). However, irrespective of the type of primary hard tissue reconstruction is simultaneous adequacy of the soft tissue reconstruction equally important: only a well vascularized soft tissue envelope allows for a successful hard tissue reconstruction. Even large series of mandibular reconstruction after ablative surgery or major trauma in literature show that oral rehabilitation of patients with large segmental defects is still a challenge [1-4]. Besides soft tissue inadequacies due to ablative procedures and scarring, the method of bony reconstruction plays an important role on the functional and aesthetic result [2,5-8]. Preferred donor sites for vascularized bone with good survival rates are the fibula, iliac bone and scapula [9-11]. Limitations in terms of either primary or secondary dental implant-based rehabilitations are especially the lack of recreating the alveolar height and following adverse crown-implant ratio. But also the lateral and sagittal extension of the bone volume determines the success rate of dental implants and therefore is crucial for oral rehabilitation in cancer patients [12]. Some authors prefer secondary mandibular reconstructions with free bone grafts which provide adequate volume to insert implants [12-14]. A prerequisite to apply this method is a secure vascularized soft tissue bed.

Pre-operative planning is highly-developed [15-17] and image-guided surgery well-established. An ongoing issue in planning procedures is that virtual reconstruction is based on a true-to-original outer contour reconstruction [18]. However, dimension limitations of bone grafts, especially in height and width, do not match with prosthodontic needs and often result in unavoidable malpositioned dental implants with sequentially lateral cross-bite situation. Furthermore, dysbalance in soft-tissue-to-bone-to-implant relationship result in later periimplantatis with consecutive early to late implant loss. Aim of this study is to implicate the ideal, i.e. prosthodontic driven planning and positioning of bone grafts in the planning procedure respecting the desired implant position: backward planning independent of the reconstructive technique. This requires patient specific pre-bending of the reconstruction plate to guide the bone graft into the desired position. So the outer contour of the mandible is not anymore the basis for bending the plate.

## Materials and methods

Three patients with tumors involving the mandible, which included squamous cell carcinoma in the floor of the mouth and keratocystic odontogenic tumor, were treated surgically by hemimandibulectomy. Written informed consent was obtained from the patient. The patient's preoperative demographic data are summarized in Table 1.

**Table 1:** Patient's preoperative demographic data

Case	ID	Age	Sex	Pathology	Planning modality
**1**	LF	53	M	Keratocystic odontogenic tumor	Mirroring of the unaffected site, STL-modell, prebending
**2**	NK	52	M	SCC (T4)	Autosegmentation, STL-modell, prebending
**3**	MB	66	F	SCC (T4)	Backward-Planning using Autosegmentation, SmartShaper, virtual Implant positioning

SCC: Squamous cell carcinoma

### Virtual Planning Technique

The imaging and planning platform used in this study was iPlan 3.0 (Brainlab^®^, Feldkirchen, Germany). This platform allows for alignment of the DICOM data set, viewing and analysis and provides 3D tools for the planning procedure such as mirroring tools, autosegmentation with atlas-function and a three-dimensional modification tool named SmartShaper. iPlan enables import of different imaging modalities and allows to automatically image-fuse these data. That facilitates not only the pre-operative analysis of the data but also the post-operative quality control. Furthermore this platform allows for intra-operative navigation of the virtual plan which is important for midface and skullbase procedures, however, this was not used in the presented cases.

The basic data used for the virtual planning included the pre-operative computed tomography (CT) imaging data with a maximum slice thickness of 1 mm. The data were transferred on the computer and reconstructed into 3D images. Different planning modalities include mirroring tools (case 1), autosegmentation tools (case 2) and the backward planning tool (case 3). The backward planning is mainly based on size-variable ideal dental arch models with adjustable and truly designable virtual hollow cylinders. These hollow cylinders define not only the implant position but also the needed peri-implant bone volume. Thus desired bone position as a required subvolume within the later bone grafts for later insertion of dental implants could be visualized and already considered during the primary alloplastic reconstruction of the mandible.

Outcome of the virtual planning is in each case a virtual template of the mandible (STL-file format) for production of a corporeal stereolithographic model. After prototyping of the model (Phacon GmbH, Leipzig, Germany) a conventional 2.4 Compact UniLOCK reconstruction plate (Synthes^®^, Umkirch, Germany) was bent to bridge the prospective mandible defect.

After ablative surgery was performed, the preformed reconstruction plate was used for the osteosynthesis. At the time of secondary reconstruction with free iliac bone graft the individual reconstruction plate gives the surgeon a clear direction where the bone should be ideally placed.

The following case studies demonstrate the planning possibilities, firstly starting with an unilateral defect using mirroring tools and patient specific reconstruction plate (case 1), secondly using the autosegmentation to virtual reconstruct the mandible (case 2) and finally presenting the prosthodontic driven "backward planning" for optimized bone graft position (case 3).

### Case Studies

#### Case 1

**Figure 1:**
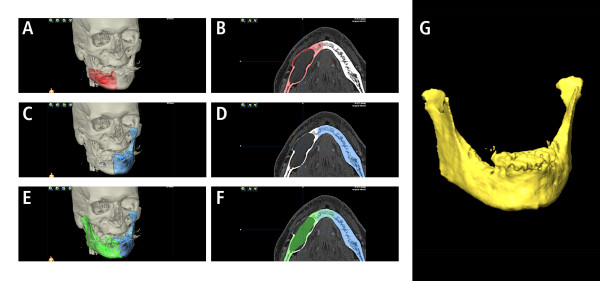
**CT scan showing an extended mandibular lesion (keratocystic odontogenic tumor)**. Swelling of the mandible makes conventionally bending prior to resection difficult (A, B). By Computer assisted pre-operative planning by mirroring of the unaffected left side (template C, D; mirrored template E, F) a stereolithographic model (G) could be printed and a reconstruction plate could be bended prior to surgery.

The patient was a 53-year-old man with a large keratocystic odontogenic tumor confirmed by previous biopsy. Due to the huge extent of the lesion, there was an indication for hemimandibulectomy of the right side. After importing the data into the imaging and planning platform (iPlan 3.0) a virtual model was built by using mirroring tools (Figure 1). The unaffected left side was manually segmented and, after alignment of the data set, mirrored to the affected site. Using logical operations "union" in the advanced manipulation mode, mirrored and unaffected left side were grouped and exported as an STL-file. Stereolithographic model was fabricated and reconstruction plate bent.

**Figure 2:**
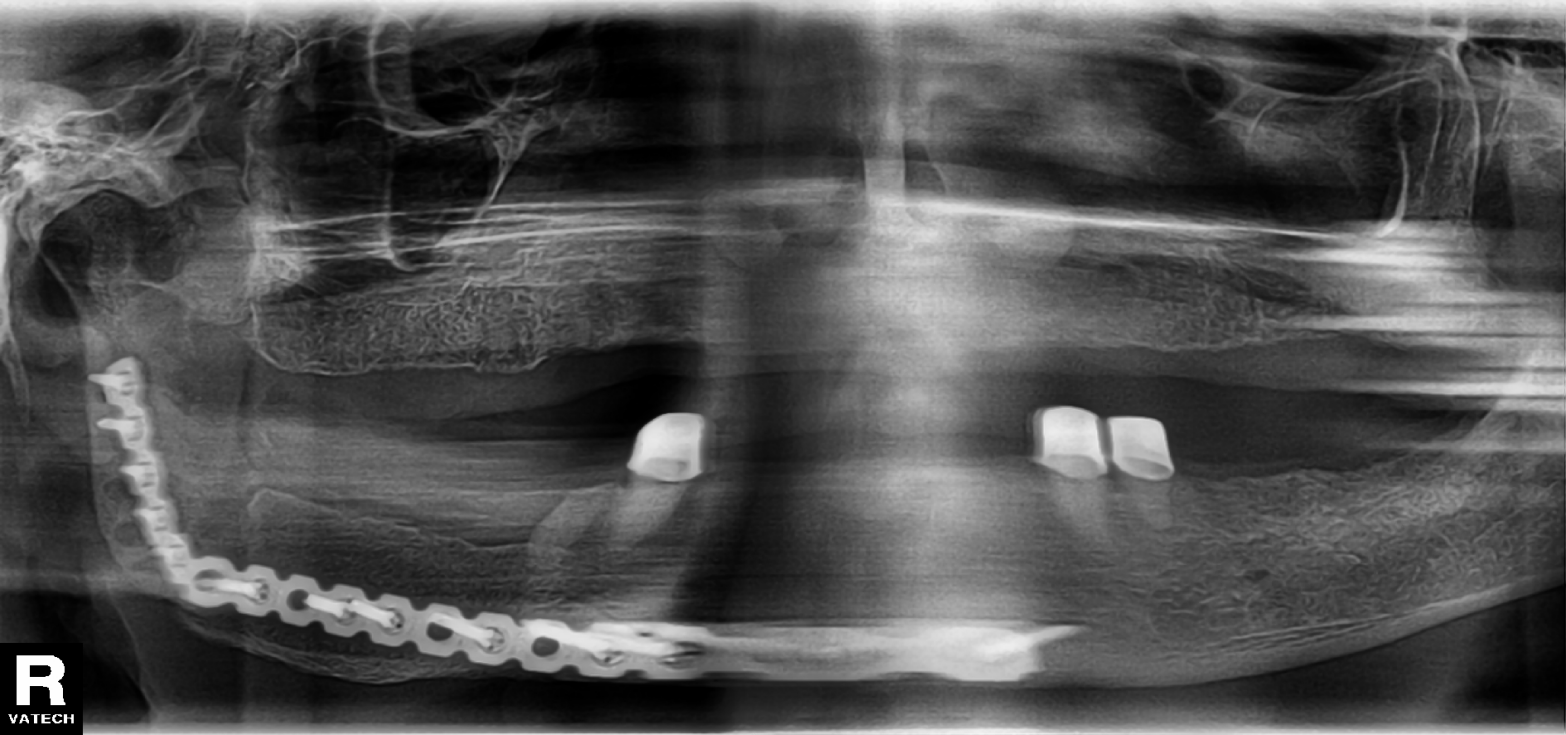
**Panorex X-ray Case 1 - after secondary reconstruction of the mandible**.

After ten months patient underwent secondary reconstruction with bone graft from the iliac crest. The operation was straightforward and quick, with no intra-operative complications. In the 2D-analysis of the panorex X-ray (Figure 2) augmentation seems adequate.

#### Case 2

**Figure 3:**
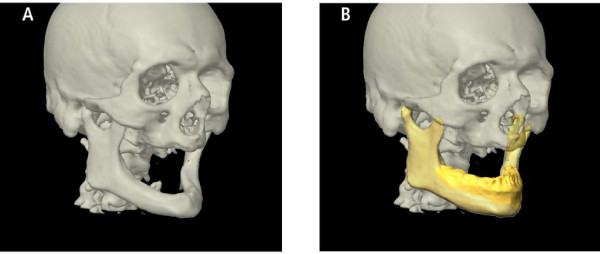
**Even in edentulous patients with severe atrophy of the mandible (A), Computer assisted planning is possible due to the autosegmentation (B) provided by the iPlan 3.0 software (BrainLAB^®^)**.

A 52-year-old male presented with a squamous cell carcinoma of the anterior floor of the mouth (pT4 N2 - Stage 4 UICC). The extent of the primary tumor and the nodal involvement was evaluated clinically and radiologically (CT scan). The expected size of mandible resection was defined virtually. Because of arrosion of the mandible and the large extent of the resection (complete corpus mandibulae), mirroring is no option for virtual planning. Brainlab iPlan 3.0 comes with an autosegmentation mode. Starting the procedure of autosegmentation, the so generated neomandible combines the existing bony structures with averaged segments of the autosegmentation. Following 3D analysis of the neomandible, a stereolithographic model was built and, as above mentioned, prebending performed (Figure 3).

Patient underwent en bloc tumor resection with partial mandibulectomy and bilateral neck dissection. The individual prebent reconstruction plate was inserted. The operation went without complications and secondary reconstruction is planned. Plate profile created good visual appearance of the lower third of the face. Besides of possible intraoral soft tissue deficiencies, large bone grafts which offer especially large dimension in width and height would be needed to enable adequate oral implantation and to prevent ending up in a cross-bite situation.

#### Case 3

The patient was a 66-year-old female who complained of dysphagia and pain caused by a tumor located on the alveolar ridge with infiltration of the anterior floor of the mouth and tongue. Diagnosis of squamous cell carcinoma was confirmed by preoperative biopsy (pT4 - Stage 4 UICC).

**Figure 4:**
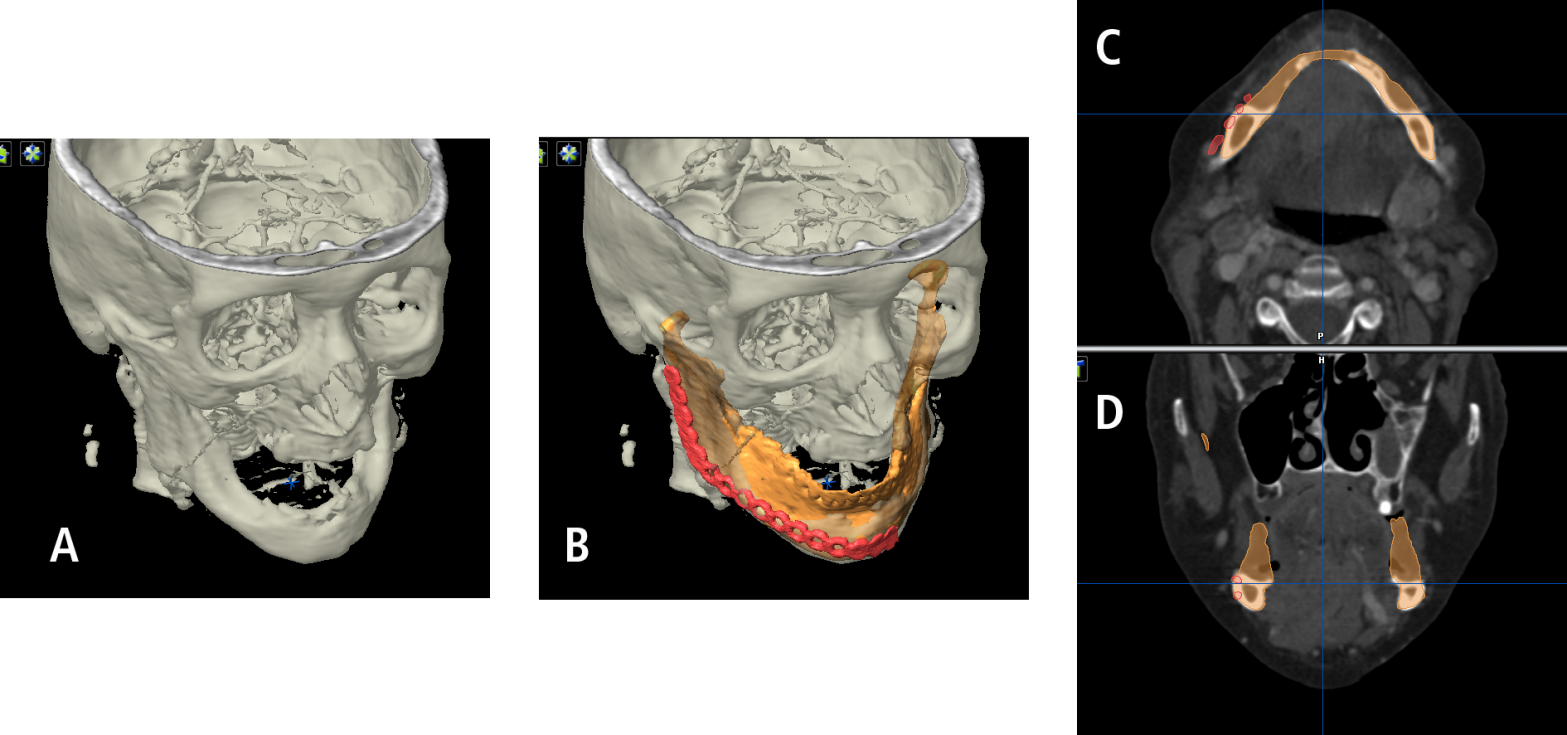
**Patient with squamous cell carcinoma facing subtotale mandibulectomy (A)**. Conventional bending of the reconstruction plate could be simulated (B). Virtual planning of the mandible by autosegmentation (similar to Figure 3) shows the conventional relation and position between plate and favored mandible reconstruction (C, D). Conventional bending of the plate follows the outer contour of the original mandible.

**Figure 5:**
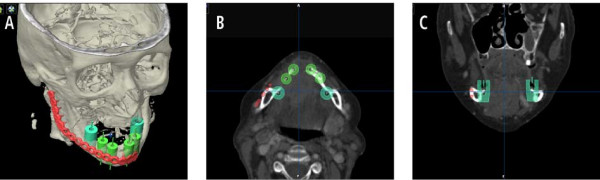
**Pre-operative planning allows prior to surgery a prosthetic-driven "backward planning"**. Even in iliac crest bone transplants reconstruction size is limited. The position of future implants with their requirements of adequate bony lining is considered (A) and simulated with different hollow cylinders. Distance between conventional position of reconstruction plates (visualized by the red plate) aligned by the original outer contour is demonstrated in two different planes (B, C).

Similar to case 2, virtual mirroring as a planning tool was not feasible due to mandible destruction and atrophy. For the autosegmentation mode, atrophy and destruction has only minor effects on template building. After autosegmentation of the mandible with iPlan, backward planning by using virtual ideal upper and lower dental arches and virtual hollow cylinder as placeholders was performed (Figure 4). The hollow cylinder illustrate a total of six dental implants and their expected requirement of bone: in this planning 3 to 4 mm bone volume was radially disposed around the cylinder (Figure 5). The diameters of implants were set at 4 mm respectively 5 mm in the posterior region. The length of the cylinders is arbitrary and was in this case between 13 and 15 mm.

**Figure 6:**
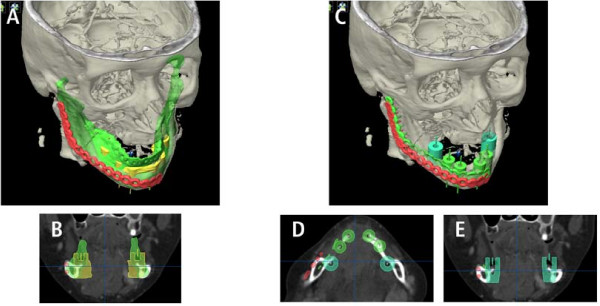
**Modified neomandible (green, A) respects the future implant position (B) and guides the new position of the reconstruction plate (green plate, C)**. The new position is mostly lingual bound (D, E).

The result of placing these hollow cylinders illustrated the target (neomandible) which should be addressed with the bone graft in secondary reconstruction. Using the SmartShaper the virtual mandible is adjusted to the desired outline of the planned neomandible and exported as STL-file (Figure 6).

## Results

In primary alloplastic mandible reconstruction, shape and size of the reconstruction plate could be predefined and prebent prior to surgery. This could improve accuracy of the contour of the plate. In limited mandible defects, good results could be achieved with this simple planning method (case 1). In larger defects (case 2) simple mirroring tools are not feasible. The recent iPlan-Software allows by using the autosegmentation mode a virtual reconstruction of the mandible and following prebending of plates.

The base of planning is up to now mostly the outer contour of the mandible. But in large reconstructions, this leads to an inappropriate positioning of bone grafts in secondary reconstruction due to the limited dimensions of bone grafts. In case 3 we could demonstrate the significant discrepancy between original mandible contour and desired neomandible contour regarding later oral rehabilitation.

## Discussion

Adequate reconstruction should offer replacement of dentition and provide improved deglutition, mastication, and speech [19] and therefore significantly contributes to oral rehabilitation. These clinically relevant parameters depend mainly on soft tissue lining, on the mobility of the tongue and on the amount of bone available for dental implantation.

Soft tissue lining has to be addressed at least twice: in mandibular reconstruction to allow a sufficient blood supply for the transplanted bone with its bradytrophic character and before or after implant insertion to allow a non-irritant interface between mucosa and implant. It is reported that in accompanying soft tissue deficiencies mandibular reconstruction is preferably treated with vascularized bone grafts. Buchbinder et al. demonstrate that osseocutaneous flaps offer the most rapid rehabilitation [19]. In contrast, there is evidence for a better outcome in oral rehabilitation of non-vascularized iliac crest grafts compared to osseocutaneous flaps [12].

Problems of bony reconstruction include undercorrection of the lower facial contour, unsatisfying midline match and unfavourable sagittal maxillary-mandibular relationship [12] due to limited bone graft volume. In literature, however, bony deficiency in width and height as an important factor for oral rehabilitation is rarely mentioned [2] and even publications which focus on virtual planning in large mandible reconstruction do not provide post-operative three-dimensional imaging [17] to make advantage of virtual planning comprehensible. Implant insertion should meet a sufficent bony base for rehabilitation [14].

There are many reports about virtual planning of mandible reconstruction. Virtual planning appears to have positive impact on the reconstruction of major mandibular defects [18]. There are commercially available planning platforms which offer planning support via web sessions [18] and also multiple research-and-development tools that allow for example as well for rapid prototyping [17,20]. The recent iPlan-Software (Brainlab AG, Feldkirchen, Germany) is a highly sophisticated commercially available product which eases planning procedures by autosegmentation and three-dimensional manipulation tools. Export function for STL-files enables rapid prototyping and intra-operative image guidance allows for computer-assisted surgery. Post-operative quality control is automatically done by superimposing pre-operative data sets including the 3D-planning with the post-operative scan.

The recent software version iPlan 3.0 allows for the described workflow in backward-planning of an ideal neomandible. A substantial limitation in commercially available planning platforms, at the moment, is the lack of an automated collision detection to find an ideal occlusal position with maximum intercuspidation. In edentulous mandible this is of subordinate importance and simplification by virtually rotation the mandible in the desired position seems to be admissible. Further software releases should address these limitations.

Bending of reconstruction plates using 3-dimensional stereolithographic models is reported. These stereolithographic models are based on virtual templates which were standardized exported in STL-file format and produced by specialized companies [15,21]. The so bended reconstruction plates offer reliable accuracy [22,23] and could ease as manually bended or as patient-specific implants not only alloplastic mandible reconstruction but also could improve placement of bone grafts to achieve an adequate bony base for later dental implantation. The backward-planning workflow could help to improve oral rehabilitation. There are still reasons for remaining without dental rehabilitation.

### Clinical relevance

This study provides modern treatment strategies for mandibular reconstruction.

## Conflict of interest statement

The authors declare that they have no competing interests.

## Authors' contributions

HE, MR, HK, CS, MRU, FT and NCG conceived of the study and participated in its design and coordination. HE and MR made substantial contributions to data acquisation and conception of manuscript. HE and MR drafted and designed the manuscript and contributed equally to this work. NCG and MRU were involved in revising the manuscript. All authors read and approved the final manuscript.

## Consent statement

Written informed consent was obtained from the patient for publication of this case report and accompanying images. A copy of the written consent is available for review by the Editor-in-Chief of this journal.

## Funding

The article processing charges are funded by the Deutsche Forschungsgemeinschaft (DFG), "Open Acess Publizieren".
